# Implementation of the Extension for Community Healthcare Outcomes Model for Hypertension Education of Frontline Health Care Workers in the Federal Capital Territory, Nigeria: Explanatory Sequential Mixed Methods Evaluation

**DOI:** 10.2196/66351

**Published:** 2025-04-24

**Authors:** Abigail S Baldridge, Adaora Odukwe, Olabisi Dabiri, L Nneka Mobisson, Maria Moosa Munnee, Ayoposi Ogboye, Dorothy Naa Korkoi Aryee, Rodrick Mwale, Jonas Akpakli, Ikechukwu A Orji, Rosemary C B Okoli, Nanna R Ripiye, Dike B Ojji, Mark D Huffman, Namratha R Kandula, Lisa R Hirschhorn

**Affiliations:** 1 Feinberg School of Medicine Northwestern University Chicago, IL United States; 2 Havey Institute for Global Health Feinberg School of Medicine Northwestern University Chicago, IL United States; 3 mDoc Healthcare Lagos Nigeria; 4 University of Abuja Teaching Hospital Abuja Nigeria; 5 University of Nigeria Nsukka Nigeria; 6 University of Abuja Abuja Nigeria; 7 The George Institute for Global Health Sydney Australia; 8 Washington University in St Louis St Louis, MO United States

**Keywords:** hypertension, implementation, primary care, education, Kirkpatrick model

## Abstract

**Background:**

The Extension for Community Healthcare Outcomes (ECHO) model was adapted for hypertension education of community health extension workers in the Federal Capital Territory, Nigeria, and delivered as a 7-part series.

**Objective:**

This study aims to evaluate implementation outcomes of the hypertension ECHO series mapped to the first 3 levels of the Kirkpatrick model. Outcomes included reach, appropriateness (level 1), effectiveness (level 2), and penetration (level 3).

**Methods:**

From August 2022 to April 2023, 7 hypertension ECHO sessions were delivered via Zoom (Zoom Video Communications, Inc) to a health care worker audience including targeted community health extension workers at 12 primary health centers (PHCs) in the Hypertension Treatment in Nigeria Program. Health care workers provided demographic information, engaged in pre- and postsession knowledge quizzes, and shared feedback during live sessions. Surveys were sent to health care workers at 12 PHCs approximately 1 month after each session to ask about the use of the presented material and focus group discussions were performed with these health care workers after the ECHO program concluded. Qualitative and quantitative results were evaluated using an explanatory sequential mixed methods design wherein qualitative data were used to help explain outcomes and variability among participants.

**Results:**

Across 7 ECHO sessions, a total of 1407 live participants were documented. Participants largely found the program was acceptable, with more than 97% of respondents reporting that the session was useful. Postsession knowledge scores increased (range: 2.3%-10.5%) relative to presession scores demonstrating moderate effectiveness. Among 12 PHCs, most (more than 70%) health care workers applied information learned in each session to provide patient care. In 6 focus group discussions, with 31 health care workers (n=15; 48% community health extension workers), participants reported that network connectivity and clinical demands were barriers to live participation and expressed preferences for blended training and asynchronous resources.

**Conclusions:**

Results show that a hypertension ECHO program adapted for community health extension workers effectively increased knowledge among participants and was useful to a majority. Insights gained may inform the scaling of remote hypertension education programs for community health extension workers in similar settings.

**Trial Registration:**

ClinicalTrials.gov NCT04158154; https://clinicaltrials.gov/ct2/show/NCT04158154

## Introduction

In Nigeria, where hypertension prevalence ranges from 29%-38%, health care workers face challenges in effectively managing this condition due to limited awareness, outdated practices, and inadequate training [1,2]. Building health care worker capacity in hypertension treatment is crucial for better care, enhanced patient outcomes, and reduced cardiovascular disease burden [3,4]. The University of New Mexico’s Extension for Community Healthcare Outcomes (ECHO) Project is an intervention aimed at empowering health care workers. Using telehealth technology, ECHO programs connect specialist medical providers with frontline health care workers, particularly in underserved and remote areas [5,6]. ECHO sessions incorporate case-based learning and bidirectional knowledge sharing with a community of practice model to facilitate longitudinal networking. The ECHO model has been used to enhance health care workers’ capacity across various areas, including hypertension [7], diabetes [8], pain management [9-15], dementia [16-18], mental health [19,20], women’s health [21], and HIV or AIDs [22]. However, there is limited implementation and evaluation of the ECHO model among community health extension workers compared to the expanding scope of their work [23-26].

Various evaluation methodologies, including pre-post studies, control group comparisons, surveys, focus group discussions (FGDs), interviews, or a combination of these, have been used to assess the impact of ECHO programs [27]. Pre-post designs mainly gauge provider self-efficacy, confidence, and knowledge improvement before and after sessions [7-10,19,20]. Qualitative assessments, through postintervention surveys and interviews, evaluate satisfaction, overall experience, program impact, and perceived benefits [15,17-19,21,22]. FGDs in some studies assessed self-efficacy, knowledge acquisition, perceived advantages, limitations, barriers, and facilitators [10,11,14]. While a recent scoping review found moderate use of mixed methods to evaluate ECHO programs, we are aware of only 2 studies that have used mixed methods to evaluate ECHO programs implemented for community health extension workers [24,26]. Robust evaluation across multiple domains through mixed methods may allow a more comprehensive understanding of outcomes and provide further contextualization for others who may be interested in implementing similar programs.

In this study, the Hypertension Treatment in Nigeria (HTN) Program team partnered with mDoc Healthcare, a digital health social enterprise specializing in ECHO, to implement and evaluate a hypertension ECHO program for community health extension workers in the Federal Capital Territory, Nigeria. In this report, we evaluated the implementation outcomes of the ECHO program mapped to the Kirkpatrick model, a well-known framework for the evaluation of learning programs [28]. We evaluated selected implementation outcomes through an explanatory sequential mixed methods evaluation which allowed comprehensive evaluation and identification of specific contextual factors associated with our selected implementation outcomes [29].

## Methods

### Study Setting, Design, and Sampling

Development of the hypertension ECHO program for community health extension workers in the Federal Capital Territory followed the ADAPT-ITT framework and has been previously described [30,31]. In our formative work, we found that the ECHO model was both feasible within the proposed setting and perceived as appropriate by health care workers for ongoing hypertension education. In this study, we report on the implementation of the ECHO program including the evaluation of implementation outcomes mapped to the Kirkpatrick model [28,32]. Selected implementation outcomes (Kirkpatrick level) included reach, appropriateness (level 1), effectiveness (level 2), and penetration (level 3; Table 1) [32]. The fourth level of the Kirkpatrick model, results, has been reported on separately through the evaluation of patient and clinic-level outcomes [33].

**Table 1:** Implementation outcomes mapped to the Kirkpatrick model for evaluation of the hypertension ECHO^a^ series.

Implementation outcome		Reach		Appropriateness		Effectiveness		Penetration
Kirkpatrick level		N/A^b^		Level 1: reaction		Level 2: learning		Level 3: behavior
**Quantitative evaluation**								
	Methods	Registration survey	Reaction survey		Pre- or postsession knowledge quizzes		Behavior survey	
	Sample	Registrants for each ECHO session	Participants from each live ECHO session		Participants from each live ECHO session		All ECHO participants at 12 HTN^c^ Program PHCs^d^	
	Outcomes	Number of participants in the ECHO program. Characteristics of participants and variation in demographics, education, and experience. Number and proportion of sessions attended among targeted health care workers.	Appropriateness of the training in terms of timing, content, and structure.		Change in hypertension knowledge among hypertension ECHO program participants.		Use of the training content in clinical practice.	
**Qualitative‍ evaluation**								
	Methods	Focus group discussion	Focus group discussion		Focus group discussion		Open-ended survey responses Focus group discussion	
	Sample	ECHO participants at 12 HTN Program PHCs	ECHO participants at 12 HTN Program PHCs		ECHO participants at 12 HTN Program PHCs		ECHO participants at 12 HTN Program PHCs	
	Outcomes	Reasons for variation in participation in the hypertension ECHO program among health care workers at selected PHCs.	The perception among health care workers among PHCs in the Federal Capital Territory, Nigeria that the hypertension ECHO program is an appropriate intervention for hypertension education.		Effectiveness of the ECHO program as platform for hypertension training among health care workers at selected PHCs.		Integration of the hypertension ECHO program among health care workers at selected PHCs into clinical practice.	

^a^ECHO: Extension for Community Healthcare Outcomes.

^b^N/A: not applicable.

^c^HTN: Hypertension Treatment in Nigeria.

^d^PHC: Primary health care center.

#### Design

**Figure 1:**
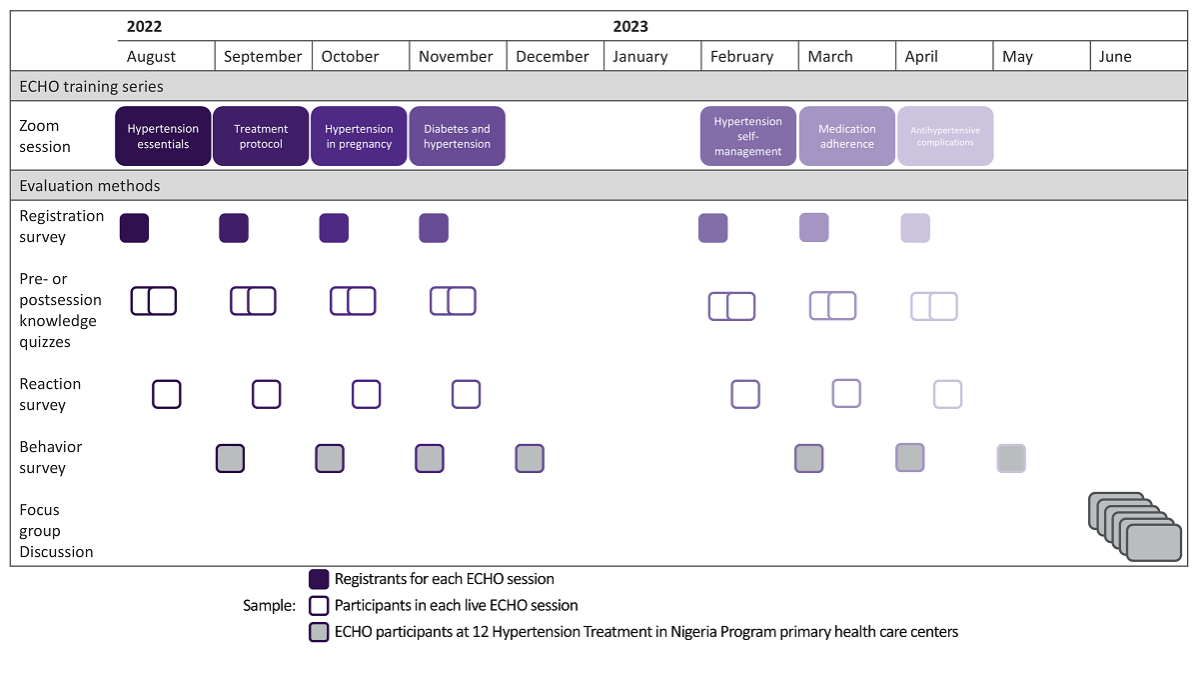
The hypertension ECHO training program and evaluation methods. The 7-part hypertension ECHO training program is depicted alongside the concurrent evaluation through Zoom (Zoom Video Communications, Inc) registration data, surveys, quizzes, and focus group discussions. ECHO: Extension for Community Healthcare Outcomes.

This study used an explanatory sequential mixed methods design to collect, analyze, and interpret quantitative and qualitative data from health care workers who participated in the hypertension ECHO series (Figure 1). The mixed methods approach was selected to broadly survey and evaluate implementation outcomes among all ECHO session participants and to confirm and expand upon the results among our target audience of community health extension workers in the Federal Capital Territory, Nigeria [29].

#### ECHO Participants

In central Nigeria, the Federal Capital Territory comprises 6 local government area (LGA) councils across which there are over 240 primary health care centers (PHCs). Sixty of these PHCs are part of the HTN Program (NCT04158154) [34]. Two PHCs from each LGA, selected as previously described, were invited to participate in the hypertension ECHO program.[31] All health care workers at these 12 PHCs were empaneled in a WhatsApp group and invited to attend each ECHO session. Sessions were advertised broadly through multiple channels including email and social media (Instagram, Facebook, and Twitter), and were open to all registered participants.

#### FGDs With Health Care Workers

Six FGDs, 1 per LGA, were conducted with health care workers from the 12 targeted PHCs. The University of Abuja Teaching Hospital (UATH) research team recruited health care workers by phone including explaining the purpose and procedures of the FGD. Health care workers who participated were all adults (aged 18 years or older), able and willing to provide informed consent, and proficient in English. We prospectively developed an FGD guide to evaluate health care workers’ reactions and experiences with the hypertension ECHO series (Multimedia Appendix 1). FGDs were conducted in English by team members trained and experienced in qualitative research (IAO and NRR). The interviewers (1 male and 1 female) were both academic researchers and primary care physicians and worked previously with the FGD participants. No translation was needed as all health care workers were comfortable speaking English. FGDs were centrally completed at UATH and each FGD comprised up to 60 minutes.

### Hypertension ECHO Program

The ECHO sessions were held approximately monthly between August 2022 and April 2023, excluding December 2022 and January 2023 due to holidays and shortened clinical schedules (Figure 1 and Multimedia Appendix 2). Every ECHO session comprised a presession knowledge quiz, introduction, didactic presentation by domain experts, a case presentation by health care workers at an HTN Program site, a postsession knowledge quiz, and a reaction survey. The mDoc Healthcare team worked with a selected HTN Program Site before each session to select and present a case for each ECHO session. Prospective participants registered in advance for each session using an web-based form that included demographic characteristics. The registrant was also asked if multiple persons planned to share the device and the total number of individuals who planned to do so. A unique link for the live Zoom (Zoom Vidro Communications, Inc) session was sent to each registrant. Reminders were sent by email to registrants 3 days, 2 days, 1 day, and approximately 3 hours before each session. Reminders were also sent on the day of the session via WhatsApp channels.

Each unique login to a Zoom session was counted as a single live device and registration data were used to account for device sharing. Waiting rooms were used to manage entry and sessions were monitored to minimize disruptions. Sessions were also live-streamed on Facebook and recordings were uploaded for public access on YouTube. Slides and recordings were sent by email to all registrants following each ECHO session.

### Outcomes

Implementation outcomes were evaluated through FGDs and survey responses, including the following.

- Reach: Registration surveys and in-Zoom tracking were used to quantify the number of ECHO program participants, the number of sessions attended among survey respondents, and the characteristics of participants including demographics, education, and experience. FGD participants provided further qualitative feedback on reasons for variation in participation in the ECHO program.
- Appropriateness (Kirkpatrick level 1): Surveys to quantitatively evaluate the appropriateness of the ECHO program in terms of timing, content, and structure were developed by the study team (Multimedia Appendix 3). Reaction surveys were administered following each ECHO session through Zoom to all live participants. FGD participants provided further qualitative feedback on the appropriateness of the ECHO program for hypertension education.
- Effectiveness (Kirkpatrick level 2): Knowledge quizzes were uniquely developed for each ECHO session by the didactic lecturers (Multimedia Appendix 4). Each knowledge quiz was administered twice through Zoom to all participants at the beginning and end of the ECHO session. The Zoom platform allowed tracking of pre- and postsession knowledge quizzes to individual devices, which allowed us to evaluate the quantitative change in hypertension knowledge among ECHO program participants. FGD participants provided further qualitative feedback on the effectiveness of the ECHO program for hypertension education.
- Penetration (Kirkpatrick level 3): Behavior surveys were developed by the study team and included questions about the application of learning within clinical practice (Multimedia Appendix 5). Behavior surveys were sent approximately 30 days following each ECHO session as a REDCap (Research Electronic Data Capture; Vanderbilt University) link through a WhatsApp message to health care workers at the 12 targeted PHCs [35,36]. FGD participants provided further qualitative feedback on the use of the training content in clinical practice and integration of the hypertension ECHO program.

### Data Management and Analysis

Survey data for reaction and learning were maintained through an enterprise Zoom account. Survey data for behavior were collected through REDCap. Quantitative data were summarized as descriptive statistics. Chi-square and Kruskal-Wallis tests were used to evaluate differences across ECHO sessions. Differences in pre-and postsession knowledge questions among respondents who participated in both sessions were compared using paired *t* tests (2-tailed) or Wilcoxon sign rank tests depending on the parametricity of the data. Statistical results were prepared using SAS (version 9.4; SAS Institute) and R (version 4.0; R Foundation for Statistical Computing). FGDs were audiotaped, transcribed verbatim, and read several times. A codebook was subsequently developed for deductive coding based on the interview guide and focused on responses related to selected implementation outcomes of reach, appropriateness, effectiveness, and penetration. The codebook was developed by a single team member (ASB) and reviewed with the full team. FGDs were analyzed thematically by a single reviewer (ASB) using Dedoose software (version 8; Socio-Cultural Research Consultants). Data integration comprised matching qualitative and quantitative results by implementation outcome through narrative connection [29]. The deidentified nature of survey and quiz responses did not allow for the direct merging of these data. Integration of the results focused on increasing the depth of understanding of the quantitative data and exploring variability among participants.

### Ethical Considerations

The reporting of this study adheres to the Consolidated Criteria for Reporting Qualitative Research (COREQ) guidelines (Multimedia Appendix 6) [37]. The protocol for surveys and FGDs with ECHO participants was reviewed and approved by the ethics committee at UATH (UATH/HREC/PR/2021/011/015) and determined to be exempt by the Northwestern University institutional review board (STU00216041).

Informed consent was not required or collected for individuals participating in the ECHO program. Registration information collected before the ECHO sessions was inclusive of individual identifiers which were removed prior to analysis. All survey data collected during the Zoom sessions were deidentified prior to analysis. ECHO session participants were not compensated. All FGD participants provided written informed consent to participate and for audio recording. Data captured in REDCap and in FGDs were unlinked to individual identifiers. Each health care worker who participated in an FGD received a 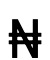 2000 honorarium (At the time, the 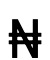 was about US $4.5 per person) plus transportation costs.

## Results

### FGDs With Health Care Workers

Across 6 FGDs, 31 health care workers participated (range: 4-7 health care workers per FGD) and no prospective participants declined (Multimedia Appendix 7). The median (range) age was 40 (26-57) years and over half (n=19; 63%) were female. Nearly half the participants (n=15; 48%) were community health extension workers and all were affiliated with the HTN Program and reported providing hypertension patient care.

### Reach

Registration was required for each session. Across all 7 sessions, 1634 registrations were submitted of which 944 (58%) registrants participated live. Accounting for device-sharing, 1407 live participants were documented. Concurrent posts on Facebook or Instagram received 5231 impressions and reached 6341 people over the 7-part series. The same posts on LinkedIn received 2966 impressions.

**Figure 2:**
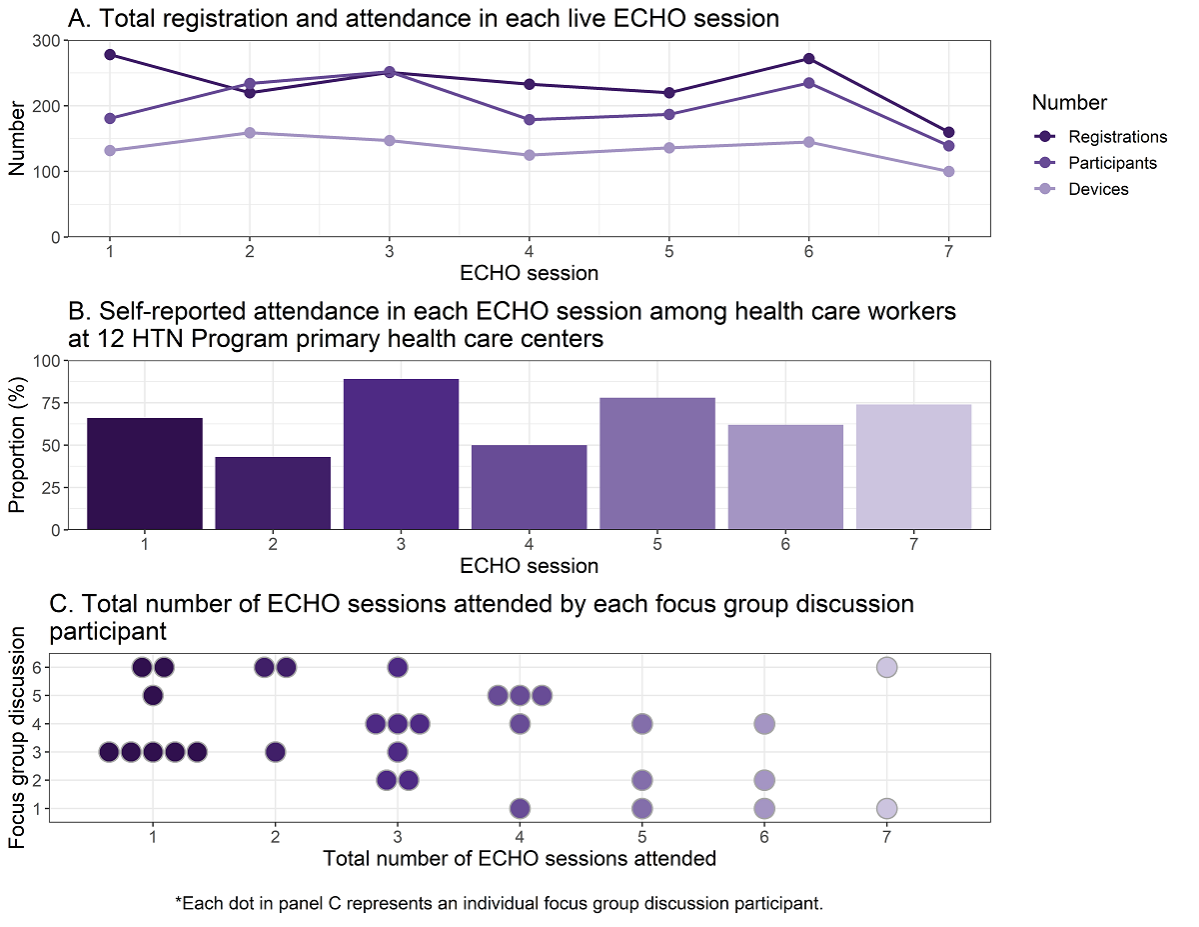
Reach of the hypertension ECHO training program. The reach of the hypertension ECHO training program was evaluated through multiple mechanisms, including registration and participation in the sessions, self-reported attendance among surveyed health care workers, and self-reported attendance among focus group discussion participants. ECHO: Extension for Community Healthcare Outcomes; HTN: Hypertension Treatment in Nigeria.

The smallest reach was observed in session 7 (n=160 registrants; n=100 live devices; n=139 live participants; Figure 2). The greatest reach for registration, number of live devices, and number of live participants were observed in session 1 (n=278 registrants), session 2 (n=159 devices), and session 3 (n=252 participants) respectively. There were no significant trends observed over time for any indices of reach (all *P* values*≥*.05). Nearly two-thirds of registered participants were female (n=594, 63%) and most were between the ages of 30 and 49 years (n=521, 55%; Table 2). Physicians were the most frequent participants (n=284, 30%) followed by nurses (n=270, 29%). Registrant occupation did not differ (*P*=.35) across sessions.

In FGDs, the health care workers discussed both barriers and facilitators to participation, which limited reach. Barriers included a lack of electrical power, an unstable network, and competing clinical demands.

Sometimes when the sessions would be fixed, my battery would be low at that time. I don't have a power bank to recharge my battery and start attending too.

FGD1, respondent 2

The network poses a serious challenge in my area. Sometimes when I attempt to connect, I will just be seeing the video without any voice or sometimes, it will not connect at all. But the three times I was able to, we had a good network on those days.

FGD3, respondent 6

When you are on the program, a patient will come in and you won't be able to continue because you have to attend to the patient.

FGD1, respondent 1

Facilitators included frequent reminders through numerous channels**—**though not all health care workers received these. Health care workers emphasized that they preferred to receive reminders through phone-based means (ie, WhatsApp) as opposed to email. Health care workers also emphasized their internal drive for knowledge as facilitators of attendance.

When it is tomorrow, we would be reminded through our emails. When it is 30 minutes to the commencement, you would be sent a text message that there's 30 minutes remaining, and when it is 15 minutes. So, that really helps me personally to know that, yes, I need to do something.

FGD1, respondent 1

We want to know more… our patients usually ask us a lot of questions. Whenever there's an opportunity to learn, yes, you want to capitalize it, so you can attend to their questions.

FGD2, respondent 3

**Table 2:** Self-reported demographic characteristics and response rates of Zoom participants in each hypertension Extension For Community Healthcare Outcomes session.

Characteristic^a^		Session (number of live devices)^b^							*P* value^c^
		1 (n=132)	2 (n=159)	3 (n=147)	4 (n=125)	5 (n=136)	6 (n=145)	7 (n=100)	
**Sex, n (%)**									.83
	Female	88 (66.7)	97 (61.0)	89 (60.5)	82 (65.6)	87 (64.0)	86 (59.3)	65 (65.0)	
	Male	44 (33.3)	62 (39.0)	58 (39.5)	43 (34.4)	49 (36.0)	59 (40.7)	35 (35.0)	
**Age (years), n (%)**									.79
	18-29	28 (21.2)	46 (28.9)	40 (27.2)	35 (28.0)	38 (27.9)	38 (26.2)	31 (31.0)	
	30-49	80 (60.6)	80 (50.3)	86 (58.5)	67 (53.6)	75 (55.1)	79 (54.5)	54 (54.0)	
	50-69	23 (17.4)	33 (20.8)	21 (14.3)	23 (18.4)	23 (16.9)	28 (19.3)	15 (15.0)	
	70 and older	1 (0.8)	0 (0.0)	0 (0.0)	0 (0.0)	0 (0.0)	0 (0.0)	0 (0.0)	
**Occupation, n (%)**									.35
	Physician	50 (37.9)	58 (36.5)	42 (28.6)	37 (29.6)	31 (22.8)	40 (27.6)	26 (26.0)	
	Nurse or midwife	30 (22.7)	48 (30.2)	46 (31.3)	33 (26.4)	40 (29.4)	46 (31.7)	27 (27.0)	
	Pharmacist	6 (4.5)	9 (5.7)	6 (4.1)	3 (2.4)	5 (3.7)	10 (6.9)	1 (1.0)	
	Laboratory scientist	1 (0.8)	1 (0.6)	1 (0.7)	3 (2.4)	3 (2.2)	3 (2.1)	1 (1.0)	
	Community health extension worker	7 (5.3)	9 (5.7)	12 (8.2)	8 (6.4)	13 (9.6)	8 (5.5)	7 (7.0)	
	Others	38 (28.8)	34 (21.4)	40 (27.2)	41 (32.8)	44 (32.4)	38 (26.2)	38 (38.0)	
**Organizational setting, n (%)**									.06
	Primary care	42 (31.8)	53 (33.3)	51 (34.7)	45 (36.0)	50 (36.8)	41 (28.3)	29 (29.0)	
	Secondary care	15 (11.4)	24 (15.1)	24 (16.3)	15 (12.0)	19 (14.0)	22 (15.2)	7 (7.0)	
	Tertiary care	18 (13.6)	28 (17.6)	24 (16.3)	16 (12.8)	9 (6.6)	23 (15.9)	12 (12.0)	
	NGO^d^ or social enterprise	48 (36.4)	38 (23.9)	41 (27.9)	41 (32.8)	53 (39.0)	52 (35.9)	47 (47.0)	
	Others	9 (6.8)	16 (10.1)	7 (4.8)	8 (6.4)	5 (3.7)	7 (4.8)	5 (5.0)	
**In-Zoom evaluation responses, n (%)**									
	Reaction Survey	49 (37.1)	39 (24.5)	37 (25.2)	31 (24.8)	43 (31.6)	42 (29.0)	30 (30.0)	N/A^e^
	Preknowledge Quiz	57 (43.2)	48 (30.2)	40 (27.2)	30 (24.0)	46 (33.8)	50 (34.5)	44 (44.0)	N/A
	Postknowledge Quiz	43 (32.6)	43 (27.0)	36 (24.5)	35 (28.0)	55 (40.4)	54 (37.2)	33 (33.0)	N/A

^a^Proportions are from among those who responded during registration. Participants selected only 1 response for each field.

^b^Reported characteristics are based on the individual who registered. Multiple persons may have participated from each device.

^c^Chi-square test.

^d^NGO: Non-governmental organization.

^e^N/A: Not applicable.

### Appropriateness

**Figure 3:**
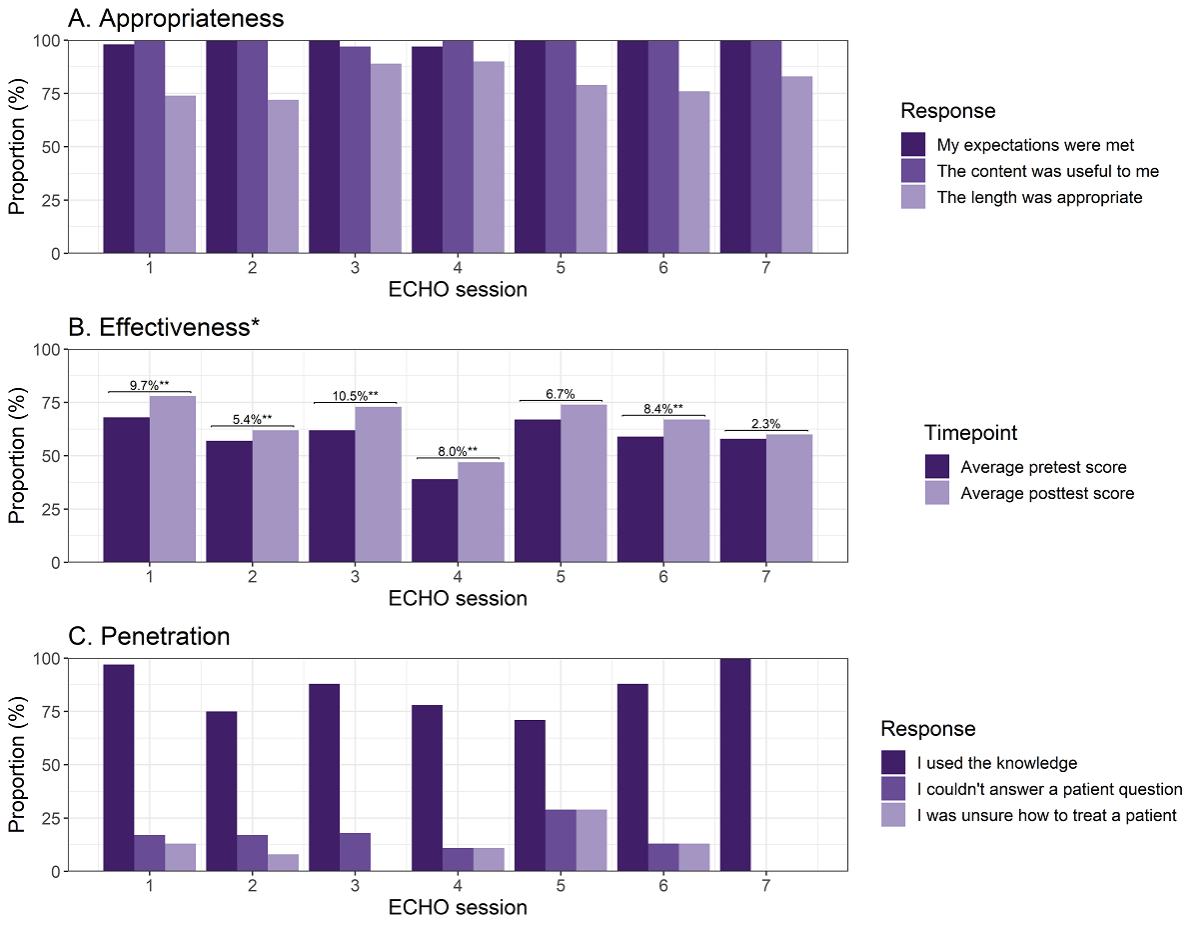
Appropriateness, effectiveness, and penetration outcomes of the hypertension ECHO training program. The appropriateness and effectiveness of the hypertension ECHO training program were evaluated through in-Zoom surveys and quizzes. Indicators for penetration of the session information were gathered around 30 days after the live offering through a REDCap (Vanderbilt University) survey among health care workers among selected primary health care centers in the Hypertension Treatment in Nigeria Program. *Bars and metrics represent the relative increase in postsession knowledge quizzes compared to the presession quiz among all attendees. ECHO: Extension for Community Healthcare Outcomes. **Denotes statistical difference (*P*<.05) based on pairwise tests between attendees who responded to both the pre- and postsession quizzes.

Nearly every participant who completed the reaction survey reported that the session met their expectations and that the training would be useful to them in treating patients with high blood pressure (Figure 3 and Multimedia Appendix 8). Most participants responded that the length of the session was appropriate (range: 72% [session 2] to 90% [session 4]). Among those who did not think the length was appropriate respondents generally felt the session was too long. In 6 out of 7 sessions, most respondents (more than 90%) reported that the didactic portion of the training was very helpful. In session 4, which focused on diabetes and pathogenesis, slightly fewer (n=22; 71% of respondents) reported that the information presented was very helpful. Respondents found case presentations slightly less helpful than didactic presentations though very few (less than 3%) reported that case presentations were unhelpful.

The qualitative results supported these results. FGD participants reported that the pedagogy and content of the training were appropriate.

The pattern of the lecture is also okay, in the sense that the lecturers will break it down. The slides are being shown, the lecturer is explaining, as well as there being a section for asking questions.

FGD1, respondent 1

It's just like the practicals we do everyday, we who see the patients. So, any time we attend the lecture, we learn from it, the challenges, and we will be picturing it, ‘Okay. Next time, if this patient comes, this is what I will do.’ That will make you want to learn more. That will make you concentrate because you are doing the practical aspect, so you want to learn.

FGD1, respondent 3

Mixed reactions were offered on the appropriateness of electronic versus in-person training. The recognized benefits of web-based training were primarily related to the convenience of location and costs.

[The ECHO Program] minimizes cost because many of us that are from far-reaching facilities, it normally takes time for us to attend training. The ECHO series has really done well by easing all that stress and the cost attached to attending meetings.

FGD3, respondent 6

Some health care workers reported a preference for in-person training, both individually and among their colleagues, citing advantages for engaging individuals’ interests, fostering interactions, and improving coverage of the workforce.

…this online stuff is not interesting…. They [colleagues] prefer a one-on-one class, instead of this ECHO stuff.

FGD2, respondent 2

Since it [training] is not an everyday thing, physical training is better because we interact with one another.

FGD3, respondent 6

Numerous health care workers advocated for electronic and physical resources to be made available before and after the sessions.

As much as we are in a digital world, there are still people who cannot operate phones like this. They cannot operate phones that would go on programs like that. So, if there is a way, maybe after the program, we can get hardcopies, that's in the form of [handouts in our facilities]. It will really help us. There are other colleagues that would want to go through them.

FGD1, respondent 3

### Effectiveness

Knowledge scores (proportion of correct responses) ranged from 39% (presession 4) to 78% (postsession 1). Across all ECHO sessions, postsession knowledge quizzes were an average of 7.3% higher than presession scores (Figure 3). The largest knowledge gain was observed in session 3 (62%-73%; 10.5%) and the smallest was observed in session 7 (58%-60%; 2.3%). Results were similar when restricted to participants who responded to both the pre-and postquiz (Multimedia Appendices 9 and 10).

FGD participants largely reported that the training was effective. Health care workers noted confidence in the accuracy of the information they received because it was coming from experts.

I would say it [ECHO] helped positively, in the sense that you are discussing with those that specialize on the topic, like the consultants that manage the condition. So, it's giving you the assurance that, indeed, you are in the right hands.

FGD2, respondent 2

The mode of presentation and then the people doing the presentation themselves, they are experts in the field. That, in a way, gives a kind of confidence that whatever they tell you is actually the right thing you're getting.

FGD4, respondent 1

The FGD participants discussed how the training directly enabled them to provide better care for their patients.

One of the sessions I attended was Improving Medication Adherence. I think that has really helped me, of course, a lot in our facility because we were having many defaulters. … So, we were able to learn how we were going to follow them up. … Some of them have made up their minds to do the right thing, and that has even improved our facility attendance, in terms of hypertension treatment.

FGD3, respondent 6

There was variability in how well participants comprehended the lectures. Some health care workers reported that the terminology used during the training was difficult for them to follow while others felt the level of information was well tailored to the audience.

I think it was more or less like it loaded more than I wished I'd known, because the depth of the ECHO series itself is quite deep. Even at some point, we were like, ‘Ah, are we in a medical class, kind of?’ Because there was a lot of depth. So, I don't think there is any information I needed that I wasn't informed about then.

FGD4, respondent 1, Data Manager

The facilitators or should I say the lecturers there, they really tried. They explained it in a way that even a layman would understand what they are saying. So, the facilitators helped a lot, and the lectures go in a way that we understand.

FGD6, respondent 6, Laboratory Technician

Actually, the background of the ECHO is very okay. It makes me concentrate on whatever I'm learning. And also, the speakers, sometimes the way they speak is with very simple English that we can understand at our own level. So, it makes me really concentrate on the session. There are not many terminologies that will– And even if there were terminologies, they usually break it down to our own level.

FGD1, respondent 2, community health extension worker

FGD participants reported that live participation in the ECHO session was key for understanding the information but that they also benefited from having access to recordings to view at a later time which made the training more effective.

Participating in that ECHO session is very key because as the lecturer is explaining the slide, you'll understand what is there... Even after the program... I would still receive another email for the video, if I want to rewatch the presentation, as well as the slides, just the slides. I used to click on both. For the videos, I do go to YouTube because they usually upload it on YouTube, so I would download it on my phone.

FDG1, respondent 1

What's exactly encouraging is that, let me say you have a network problem, there is a video there. After the program, you can still click there and watch what was being discussed there. Honestly, that one encourages me to learn more from it.

FGD5, respondent 4

### Penetration

Behavior survey results are reported only among health care workers working in the FCT, Nigeria, and were completed a median (IQR) of 28 (24-35) days after the ECHO session (Multimedia Appendix 11). Individuals who provided patient care and participated in the last ECHO session were asked about their use of the information and gaps in their knowledge. Most respondents (more than 70%) in each survey reported that they used what they learned in the immediate prior ECHO session for patient care (Figure 3). Open-ended survey responses supported the application (ie, “I learnt on how to take care of a pregnant woman with hypertension [and] also the risk factors for developing hypertension disorder in pregnancy”).

A small number (range: 0 [0%] to 2 [29%]) of respondents reported knowledge gaps at each survey point. Gaps included the lack of understanding of risk factors for hypertension (ie, “Can obesity lead to hypertension?”) and duration of drug therapy (ie, “How long are we [the patient] going to continue taking the drugs?”).

FGD participants recommended that the hypertension ECHO program should be continuous and expanded to larger cohorts inclusive of both additional health facilities and health care workers.

There should be periodic training. It shouldn't be for just particular people or a particular set in the facility alone. By the time this set is trained, another set should be called, different faces from that facility should be trained as well, so that when this person is not around, the other person is around.

FGD1, respondent 1

[My suggestion] is extending to other facilities, because as the program is going on now, I can see that selected clinics are chosen from each of the area councils.

FGD6, respondent 1

We actually need training. Science is dynamic and we keep seeing new things every day … this program has really widened our knowledge in terms of hypertension treatment, and we will be happy to see more of this training.

FGD3, respondent 6

## Discussion

### Principal Findings

We evaluated the implementation of a seven-part hypertension ECHO series to train health care workers in Nigeria through selected implementation outcomes mapped to the first 3 levels of the Kirkpatrick model. To our knowledge, this study is one of the first to use the full Kirkpatrick model for the evaluation of an ECHO program. Prior evaluations, which largely focus on domains of reaction and learning, align with our findings of high satisfaction and moderate knowledge gains [38-40]. A large majority of FGD participants reported using information from ECHO sessions in their patient-related work, which is consistent with findings from a scoping review in which 40% of the reviewed studies found that ECHO participants either modified their practice behaviors or intended to do so as a result of participation [41].

Our findings underscore the necessity for a supportive ecosystem that enables greater reach. FGD participants highlighted several barriers to live participation including busy work schedules and systemic challenges such as poor mobile networks and the unavailability of electricity to power their devices. In our implementation, we sent frequent reminders through an assortment of channels such as email, text messages, and social media platforms (WhatsApp, Facebook, Instagram, and LinkedIn). This strategy aimed to leverage health care workers’ intrinsic motivation for learning to enhance uptake, active participation, and adherence. Our approach of using multiple communication channels to promote ECHO sessions stands out from the usual practice of tailoring ECHO sessions to specific groups and enables us to reach a larger and more diverse audience.

FGD participants expressed a mixture of opinions on the appropriateness and effectiveness of the ECHO model specifically in how they thought training should be delivered. We observed preferences for a blend of in-person and web-based training with web-based training delivering both synchronously and asynchronously. In-person sensitization training could be partnered with a continuous ECHO program and a digital learning management system to promote a community of practice and facilitate access to recorded sessions and learning materials.

### Limitations

The delivery and evaluation of this intervention were subject to several limitations. First, considerable time was dedicated to providing digital and in-person support to participants and limited access to mobile data and reliable internet connections restricted the number of people that could be supported by the intervention. Unique to this intervention was the backend infrastructure required to successfully host the entire series. It was run by a consortium of partners who focused separately on recruiting speakers, facilitating the sessions, publicizing the events, and collecting and analyzing study data. Second, the inability to track individual participants across sessions meant that outcomes and assessments were only evaluated per session instead of across the entire series. Third, the use and behavior surveys relied on self-reported data, which prevented us from objectively examining the impact of the sessions on participant behavior. Despite these limitations in data collection, our approach of including all comers and robust evaluation through mixed methods provides ample data for systematic evaluation of the ECHO series and may serve as a model for evaluations of other programs.

### Conclusions

Our hypertension ECHO session resulted in substantial satisfaction and knowledge acquisition among participating health care workers. The program had wide reach despite challenges such as poor connectivity and competing clinical demands which hindered participation. Our findings underscore the importance of creating a supportive and flexible ecosystem for training and demonstrate that the ECHO program can be successfully implemented for hypertension education of health care workers in the FCT, Nigeria. This evaluation provides valuable insights for the potential scaling of hypertension education for health care workers in similar settings.

## Data Availability

The datasets analyzed during the current study are not publicly available because the data collection, as approved by the ethics committee and institutional review board, did not include having them become publicly available. The data can be made available to other researchers by contacting the corresponding author and with ethics committee and institutional review board approval.

## Appendix

Multimedia Appendix 1 Semistructured guide for focus group discussions.

Multimedia Appendix 2 Offerings, experts, and attendees of the hypertension ECHO (Extension for Community Healthcare Outcomes) training program.

Multimedia Appendix 3 Postsession zoom survey to assess reaction (Kirkpatrick Level 1).

Multimedia Appendix 4 Pre- and postsession zoom quizzes to assess knowledge (Kirkpatrick Level 2).

Multimedia Appendix 5 Postsession REDCap (Research Electronic Data Capture) survey to assess behavior (Kirkpatrick Level 3).

Multimedia Appendix 6 COREQ (Consolidated Criteria for Reporting Qualitative research) checklist.

Multimedia Appendix 7 Characteristics of frontline health care worker focus group discussion participants from 12 Hypertension Treatment in Nigeria Program primary health centers.

Multimedia Appendix 8 Reaction surveys from live participants in each hypertension ECHO session.

Multimedia Appendix 9 Pre- and postsession knowledge quizzes from each hypertension ECHO (Extension for Community Healthcare Outcomes) session among all participants who responded.

Multimedia Appendix 10 Pre- and postsession knowledge quizzes from each hypertension ECHO (Extension for Community Healthcare Outcomes) session among participants who responded to both the pre- and postsession quizzes.

Multimedia Appendix 11 Participant feedback on use of information following each ECHO (Extension for Community Healthcare Outcomes) session among health care workers at 12 Hypertension Treatment in Nigeria Program primary health centers.

## Abbreviations

COREQ: Consolidated Criteria for Reporting Qualitative research
ECHO: Extension for Community Healthcare Outcomes
FGD: Focus group discussion
HTN: Hypertension Treatment in Nigeria
LGA: local government area
PHC: primary health center
REDCap: Research Electronic Data Capture
UATH: University of Abuja Teaching Hospital
