# Supplementary material for: Implementation of the Extension for Community Healthcare Outcomes Model for Hypertension Education of Frontline Health Care Workers in the Federal Capital Territory, Nigeria: Explanatory Sequential Mixed Methods Evaluation
Source: J Med Internet Res. 2025 Apr 24;27:e66351. doi: 10.2196/66351 (PMC12062761; doi:10.2196/66351)
Supplement: Multimedia Appendix 1 [file jmir_v27i1e66351_app1.pdf]

FGD Guide

Introduction:
Thank you meeting with me today. My name is [NAME]. I am working with the Cardiovascular Research Unit of University of Abuja and University of Abuja Teaching Hospital.

Over the past year you took part in a pilot program in which you participated in learning sessions over zoom, discussed cases from PHCs in the Federal Capital Territory, and completed surveys. We would like to hear your feedback on this training program.

This focus group discussion will take about 90 minutes. In order to get your valuable comments, I will be recording the session and taking some notes as well. Your responses will be kept confidential and we will ensure that any information to be included in our report does not identify you as the respondent. Do you have any questions before we begin?

Focus Group Discussion:

1. How many of the HTN ECHO sessions did you participate in?
    a. What prevented you from participating in ECHO sessions?
    b. What helped you to participate in ECHO sessions?

2. Please describe what you learned during the HTN ECHO sessions.
    a. If nothing, please explain why you did not learn

3. How effective was the ECHO program in helping you learn more about high blood pressure?
    a. What was it about the ECHO program help you to learn?
    b. What was it about the ECHO program did not help you to learn?

4. What could be improved about this ECHO program for other CHEWs?

5. Please describe what you need in order for you and your primary health center to continue treating patients with high blood pressure
    a. How do you think training should or could be delivered?
    b. What should the contents of the training be?
    c. What more do you wish you knew?

6. How well do you think the ECHO program training you have received helps you to meet the needs of patients served by your primary health center?
    a. In what ways has the ECHO program training you have received helped you to meet your patients' needs?
    b. How could the ECHO program training be improved to help you better meet your patients' needs?

7. What kinds of incentives do you think are needed to ensure that CHEWs participate in ongoing high blood pressure training like the ECHO program?

8. What resources would you need in order to participate in training like the ECHO program going forward?
