# Supplementary material for: Implementation of the Extension for Community Healthcare Outcomes Model for Hypertension Education of Frontline Health Care Workers in the Federal Capital Territory, Nigeria: Explanatory Sequential Mixed Methods Evaluation
Source: J Med Internet Res. 2025 Apr 24;27:e66351. doi: 10.2196/66351 (PMC12062761; doi:10.2196/66351)
Supplement: Multimedia Appendix 2 [file jmir_v27i1e66351_app2.docx]

**Table S1.** Offerings, experts, and attendees of the hypertension ECHO training program.

|  | **Domain** | **Description** | **Didactic Experts** | **Date** | **No. of Registered Participants** | **No. of Live Devices^1^** | **No. of Live Participants^2^** |
| --- | --- | --- | --- | --- | --- | --- | --- |
| 1 | Hypertension Essentials | Understanding the practical approaches necessary to obtain accurate BP measurements and the various hypertension diagnostic categories and their therapeutic implications. | Dr. Okechukwu Ogah  Dr. Dike Ojji | Aug 11, 2022 | 278 | 132 | 181 |
| 2 | Guideline Based Treatment Protocol & Combination Therapy | When and how to use initial combination agents for combination therapy. How to identify and manage resistant hypertension and when to seek referral. | Dr. Kufor Osi  Dr. Mahmoud Sani  Dr. Amam Mbakwem | Sep 8, 2022 | 220 | 159 | 234 |
| 3 | Hypertension in Pregnancy | Opportunities for early intervention to reduce the health impact of diabetes in pregnancy. Understanding adverse pregnancy outcomes such as preeclampsia and eclampsia. | Dr. Malachy Emeka Ayogu  Dr. Zainab Mahmoud | Oct 13, 2022 | 251 | 147 | 252 |
| 4 | Diabetes and Hypertension: Treatment and Management | Incidence of kidney disease is likely underreported, and comorbid diabetes and obesity represent a moderate proportion of referrals. Diagnosis and management of comorbid hypertension and diabetes. | Dr. Solomon Kadiri  Dr. Brian Raynor | Nov 10, 2022 | 233 | 125 | 179 |
| 5 | Teaching Patient Hypertension Self-Management Skills | Additional training on counselling may improve triangulation of medication usage and strengthen the ability of CHEWs to provide effective self-management counselling services. | Dr. Nkechi Obianozie | Feb 16, 2023 | 220 | 136 | 187 |
| 6 | Improving Medication Adherence | Importance of adherence, patient counselling to improve adherence, methods for ascertaining adherence. | Dr. Ejiro Umuerri  Dr. Simeon Isuzu | Mar 16, 2023 | 272 | 145 | 235 |
| 7 | Complications of Antihypertensive Drug Therapy | Assessment, identification, and management of side effects. | Dr. Emmanuel Ejim  Dr. Adimbola Opadeyi  Dr. Ifeoma Ulasi | Apr 13, 2023 | 160 | 100 | 139 |

^1^Number of unique devices (laptop, phone, etc.) that joined the Zoom session. This excludes participants who joined the live session through Facebook.

^2^Number of unique participants who joined the Zoom session, accounting for instances where multiple persons joined from a single device. This excludes participants who joined the live session through Facebook.
