# Supplementary material for: Implementation of the Extension for Community Healthcare Outcomes Model for Hypertension Education of Frontline Health Care Workers in the Federal Capital Territory, Nigeria: Explanatory Sequential Mixed Methods Evaluation
Source: J Med Internet Res. 2025 Apr 24;27:e66351. doi: 10.2196/66351 (PMC12062761; doi:10.2196/66351)
Supplement: Multimedia Appendix 3 [file jmir_v27i1e66351_app3.pdf]

Reaction

Please complete the survey below.

Thank you!

Did the training meet your expectations?	◯ Yes<br>◯ No
Do you think the training will be useful to you in treating patients with high blood pressure?	◯ Yes<br>◯ No
Was the length of the session appropriate?	◯ Yes, the length was just right<br>◯ No, it should have been shorter<br>◯ No, it should have been longer
How helpful was the material presented by the speakers?	◯ Very Helpful<br>◯ Somewhat Helpful<br>◯ Neither Helpful or Unhelpful<br>◯ Somewhat Unhelpful<br>◯ Unhelpful
How helpful was the case presentation?	◯ Very Helpful<br>◯ Somewhat Helpful<br>◯ Neither Helpful or Unhelpful<br>◯ Somewhat Unhelpful<br>◯ Unhelpful
