# Supplementary material for: Implementation of the Extension for Community Healthcare Outcomes Model for Hypertension Education of Frontline Health Care Workers in the Federal Capital Territory, Nigeria: Explanatory Sequential Mixed Methods Evaluation
Source: J Med Internet Res. 2025 Apr 24;27:e66351. doi: 10.2196/66351 (PMC12062761; doi:10.2196/66351)
Supplement: Multimedia Appendix 4 [file jmir_v27i1e66351_app4.docx]

Poll Questions and Answers for the 7 HTN series sessions

***Answers for each question are in bold and italicized***

**Part 1:** HYPERTENSION ESSENTIALS

**Speakers:**

Dr. Dike Ojji, MBBS, PhD, FWACP, FACP, FESC

Dr. Okechukwu Ogah, MBBS, Msc, PhD

1. In recording blood pressure values, the following statement is true:
   1. The upper value is termed diastolic
   2. The lower value is termed systolic
   3. ***The upper value is termed systolic***
   4. The upper value is always greater than 140 mmHg
   5. The lower value is always greater than 90mmHg
2. As regards blood pressure, choose the correct statement
   1. Blood pressure is the force exerted on the veins by flowing blood
   2. Pulse pressure is the addition of systolic and diastolic blood pressures
   3. ***Hypertension is persistently elevated blood pressure***
   4. Systolic blood pressure corresponds to relaxation of the heart
   5. Diastolic blood pressure corresponds to contraction or the squeezing of the heart.
3. Which of the following is a quality of a good blood pressure machine?
   1. It is expensive
   2. It is complex to use
   3. ***It is validated***
   4. It gives inaccurate readings sometimes
   5. It should be replaced often
4. As regards blood pressure machines, the following statement is not an advantage of the digital machine.
   1. Require less training than other devices
   2. No calibration needed
   3. Does not require interpretation of sounds
   4. Does not require the use of a stethoscope
   5. ***It is difficult to use by field workers***
5. In the measurement of high blood pressure, the following precautions should be taken.
   1. Patients should be seated comfortably in a quiet environment less than 1 min before beginning BP measurements
   2. ***Patients should be seated comfortably in a quiet environment for about 5 minutes before beginning BP measurements***
   3. Patients should have their legs crossed when seated to have accurate blood pressure measurements
   4. Patients should consume some coffee 20 minutes before their blood pressure is measured
   5. The patients should be made to talk when the blood is being measured
6. The following is correct as regards the staging of hypertension
   1. Optimal blood pressure values are systolic BP less than 140mmHg and diastolic blood BP less than 90mmHg
   2. Grade 1 hypertension is systolic BP of 160-179mmHg and diastolic blood pressure of 100-109 mmHg
   3. ***Grade 1 hypertension is systolic BP of 140-159mmHg and diastolic BP of 90-99mmHg***
   4. Grade 3 hypertension is systolic BP of 200 mmHg and above and diastolic BP of 120mmHg
   5. Normal BP is greater than 150mHg systolic
7. The following statement is correct why high BP should be treated
   1. To reduce damage to the skin
   2. To reduce damage to the hair
   3. Normal blood pressure values make the patients happy
   4. ***It helps in protecting target organs***
   5. It improves memory
8. A 55-year-old man has been found to have a BP reading of 172/104. How should he be managed based on the Nigerian Hypertension protocol?
   1. Commence Amlodipine 10mg
   2. Commence Amlodipine 5mg
   3. Commence Losartan 50mg
   4. Commence Losartan 100mg
   5. ***Commence Amlodipine 5mg plus Losartan 50 mg***

**PART 2:** GUIDELINE-BASED TREATMENT PROTOCOL AND COMBINATION THERAPY (HIGHLIGHTING RESISTANT HYPERTENSION)

**Speakers:**

Prof. Mahmoud U. Sani, MBBS, PhD, FWACP, FACP, FACC, FNCS, FESC, FRCrofessoP Edin

Dr. Kufor Osi, MBBS, MPH.

Prof. Amam Chinyere Mbakwem MBBS, FWACP, FACC, FESC.

1. Treatment guidelines are comprehensive and provide multiple pathways and options for managing a condition while treatment protocols are rigid, specific in detail, and are meant to be used by all cadres of healthcare workers:
   1. ***True***
   2. False
2. Combination treatment for hypertension refers to the use of two or more medications from the same class of anti-hypertensive agents:
   1. True
   2. ***False***
3. One of the following is not an indication for combination therapy in Hypertension:
   1. Grade 2 HTN and above
   2. ***Baseline blood pressure ≥ 20/10 mmHg above target BP***
   3. Treatment of a co-morbidity e.g Angina
   4. Hypertension in blacks
4. Some advantages of combination therapy include all except:
   1. It decreases the pill burden for patients
   2. It reduces cardiovascular events in patients
   3. It promotes better long-term blood pressure control
   4. ***It decreases adherence to medication***
5. Resistant hypertension is blood pressure above goal despite optimal use of three anti-hypertensive medications of different classes including a diuretic:
   1. ***True***
   2. False
6. Criteria for referral of patients to higher levels of care include all except:
   1. BP ≥ 180/110 mmHg
   2. ***Presence of Target organ damage***
   3. Symptoms of Heart Failure
   4. Uncontrolled Hypertension
7. One of the following combinations is not part of WHO recommended combinations:
   1. CCB + ACEi
   2. CCB + ARB
   3. ACEi + thiazide diuretic
   4. ***BB + thiazide diuretic***
8. The following is true about the Nigerian Hypertension Treatment Protocol (Choose one):
   1. It is for all age groups
   2. It does not allow for referral to a specialist
   3. ***It recommends starting with Amlodipine and adding other drugs if BP is not controlled***
   4. One of the drugs recommended as add-on medication is Propranolol

**PART 3:** HYPERTENSION IN PREGNANCY

**Speakers:**

Dr. Zainab Mahmoud MD, MSC

Dr. Malachy Emeka Ayogu, MBBS, FWACS, FMCOG

1. A pregnant woman presents to you at 12 weeks gestation. She has a history of migraines. She reports intermittent headaches and her blood pressure today was 152/88mmHg. What is the most likely diagnosis:
   1. Preeclampsia
   2. ***Chronic Hypertension***
   3. Eclampsia
   4. Gestational Hypertension
   5. Normal pregnancy symptoms
2. Chronic Hypertension in pregnancy is managed as follows:
   1. Continue regular medications with no changes
   2. Stop all medications until delivery
   3. Advise the mother to terminate the pregnancy
   4. ***Review medications and use only medications safe in pregnancy***
   5. None of the above, pregnant women do not have chronic hypertension
3. A pregnant woman presents to you at 36 weeks gestation at a primary health care center with headaches and blurry vision. Her blood pressure is 142/85mmHg. She does not feel well. Your primary health care center has antihypertensive medication but no delivery services. What is the next best step:
   1. ***Refer her to the nearest obstetric emergency immediately***
   2. Prescribe paracetamol and advise rest
   3. Prescribe labetalol 200mg and discharge home
   4. Advise her to return if she starts having contractions
   5. Monitor her in the primary health care center for about 24 hours
4. Which of the following statements are false:
   1. Women >35 years old can develop preeclampsia
   2. Preeclampsia can occur without proteinuria
   3. Preeclampsia can cause maternal death
   4. Gestational hypertension is diagnosed after 20 weeks of gestation
   5. ***Women less than 25 years of age do not get preeclampsia***
5. Expectant management of hypertension in pregnancy is contraindicated in the following except:
   1. Fetal compromise
   2. Eclampsia
   3. Pulmonary edema
   4. ***Blood pressure 140/90mmHg***
   5. Cerebral edema
6. Principles of management of severe pre-eclampsia/eclampsia include the following except:
   1. Control of blood pressure
   2. Control or prevent convulsion
   3. Fluid and electrolyte management
   4. Delivery via the safest and fastest route
   5. ***Deliver by caesarean section in the absence of any other obstetric indication***
7. Which of the following is NOT advised to be used in hypertension during pregnancy?
   1. Alpha methyl dopa (Aldomet)
   2. ***Ergometrine***
   3. Oxytocin
   4. Nifedipine
   5. Dexamethasone
8. These are all true about Mso4 toxicity except:
   1. IV calcium gluconate is used as an antidote
   2. Loss of patellar reflexes is a sign
   3. Respiratory difficulty is a sign
   4. Cardiac arrest is a sign
   5. ***Abruptio placentae is a sign***
9. Which of the following is NOT a complication of severe preeclampsia/eclampsia?
   1. IUGR
   2. Preterm delivery
   3. Dissemination Intravascular Coagulation (DIC)
   4. Oliguria
   5. ***None***
10. Risk factors for preeclampsia include the following:
    1. Primigravidity
    2. Extremes of age
    3. multiple gestations
    4. ***All of the above***
    5. none of the above

**PART 4:** DIABETES AND HYPERTENSION - TREATMENT AND MANAGEMENT

**Speakers:**

Professor Solomon Kadiri, MBBS, FMCP (Nig), FWACP, FRCP (London)

Professor Brian Rayner M.B.Ch.B., MMed, and PhD

1. A 50-year-old male office worker, otherwise well, has his blood pressure taken a number of times over a 2-week period. Which of the following sets of office readings (mmHg) indicates hypertension?
   1. 136/82; 138/90; 124/82; 133/88
   2. ***148/72; 154/81; 140/81; 151/79***
   3. 142/76; 130/73; 121/78; 113/68
   4. 126/88; 115/80; 133/90; 142/83
2. Hypertension associated with type-1 diabetes typically occurs as a result of
   1. ***chronic kidney disease***
   2. hyperinsulinaemia
   3. sodium retention
   4. side effects of medications
3. What is the most likely diagnosis in a 60-year-old man with a body mass index of 32 Kg/m2 and repeated fasting blood sugar of over 7.5 mmol/L?
   1. metabolic syndrome
   2. type-1 diabetes
   3. ***type-2 diabetes***
   4. impaired blood glucose
4. The metabolic syndrome essentially differs from type-2 diabetes by the absence of the following:
   1. obesity
   2. Hyperinsulinemia
   3. Hypertension
   4. ***elevated blood sugar***
5. The prevalence of hypertension in T2DM is:
   1. 50%
   2. 60%
   3. 70%
   4. ***80%***
6. The dominant risk intervention in reducing CV events in diabetes is:
   1. Reducing HbA1C to target
   2. Treating hyperlipidaemia with statins
   3. ***Lowering BP to target***
7. In an African patient with T2DM and a BP 150/90mmHg should ideally be initiated on which of the following:
   1. ACE inhibitor
   2. CCB
   3. Thiazide like diuretic
   4. ***Combination of either ACE inhibitor or ARB with a CCB***
8. In patients, SGLUT2 inhibitors have been shown to: Select incorrect answer
   1. Lower BP
   2. Reduce weight
   3. Prevent heart failure
   4. Prevent progression of kidney disease
   5. ***Prevent stroke***

**Part 5:** TEACHING PATIENT HYPERTENSION SELF-MANAGEMENT SKILLS.

**Speakers**

Dr. Nkechi Obianozie. MBBS FWACP

Dr. Aima Uagboe. Associate Fellow NPMCN

1. Hypertension self-management strategy includes which of the following:
   1. Weight gain
   2. Water rationing
   3. Lifestyle modifications have a reduced role to play
   4. ***Combining home self-management with team care***
2. In checking blood pressure in includes the following steps except:
   1. Rest for 5 minutes before blood pressure reading
   2. Refrain from talking while testing
   3. Cuff at the level of the heart
   4. ***Legs can be crossed to increase blood flow to the heart***
3. The DASH diets:
   1. Low in potassium
   2. Rich in trans fats
   3. ***Whole grain is preferable***
   4. Nuts and seeds at least 3 servings per week
4. Patient education on self-management for hypertension includes the following except:
   1. Blood pressure targets
   2. Recording and monitoring
   3. Lifestyle modifications
   4. ***Minimal consideration for obstacles to self-management***
5. In helping members achieve their goals the following can be employed except:
   1. Motivational interviewing
   2. ***Goals should be Specific and changeable***
   3. Tracking and follow-up is key
   4. Baseline information should be taken
6. Exercise for people living with hypertension, which is incorrect:
   1. Leads to improved cardiovascular health independent of blood pressure reduction
   2. Identify strategies to enhance decision to exercise
   3. ***Minimum of 400 minutes of exercise a week***
   4. Resistance training has moderate benefits
7. On alcohol intake and smoking for people with hypertension
   1. Alcohol is totally forbidden
   2. ***Alcohol can be taken within specified amounts***
   3. Smoking is allowed but within specified limits
   4. Alcohol should be limited to 1 standard drink daily for both men and women

**Part 6:** IMPROVING MEDICATION ADHERENCE.

**Speakers**

Prof. Simeon Isezuo. MBBS, MHPE, MD, FMCP, FRCP, FESC, FNCS, FAMedS, Cert Card.

Dr. Ejiroghene Martha Umuerri. MBBS, MPH, FWACP, FMCP.

1. Which of these patients has medication adherence:
   1. A patient who uses the prescribed medication.
   2. ***A patient who uses the prescribed medication at the dose prescribed and at the time prescribed to be used.***
   3. A patient who uses the prescribed medication at the time prescribed and at a lower dose.
   4. A patient who uses the prescribed medication and completes the dosage though at a later time than prescribed.
   5. B&D
2. Which of the following is an indication for stopping antihypertensive medicines?
   1. Absence of symptoms
   2. Normal blood pressure
   3. Taking treatment for another illness
   4. Pregnancy in women
   5. ***None of the above***
3. Which of the following is correct?
   1. Primary non-adherence is when a patient decides to lower the dose of a prescribed medication.
   2. Tertiary non-adherence is when a patient decides not to purchase a prescribed medication.
   3. Secondary non-adherence is when a patient decides to lower the dose of a prescribed medication.
   4. Tertiary non-adherence is when a patient decides to stop a prescribed medication without the doctor’s advice.
   5. ***Secondary non-adherence is when a patient decides to stop a prescribed medication without the doctor’s advice.***
4. One of the methods of assessing medication adherence for patients on antihypertensives includes
   1. Pill counting
   2. Patient medication diaries
   3. Self-report survey like MMAS-8
   4. ***All of the above***
   5. B&C only
5. Most cases of medication non-adherence are intentional
   1. ***True***
   2. False
6. The following are consequences of not adhering to antihypertensive medications EXCEPT
   1. Stroke
   2. Myocardial infarction (Heart attack)
   3. Kidney failure
   4. ***Improved quality of life***
   5. Arteriosclerosis
7. The direct method of assessment of medication adherence is more effective than the indirect method among patients with hypertension
   1. True
   2. **False**
8. The following are steps to improve medication adherence among patients, EXCEPT?
   1. ***Multiple-dose regimen***
   2. Electronic reminders
   3. Involve the patient in the discussion of the treatment
   4. Check the patient’s understanding of the health education provided
   5. None of the above

**Part 7:** Complications of Antihypertensive Treatment

**Speakers**

Prof. Emmanuel Ejim. MBBS, FMCP, FESC, FACC, FNCS.

Prof. Ifeoma Ulasi. MBBS, FWACP, FRCP.

Dr Abimbola Opadeyi. MBBS, PhD, FWACP, FMCP.

1. Side effects are the same as toxic effects
   1. True
   2. **False**
2. What the body does to the medicine is called Pharmacokinetics
   1. **True**
   2. False

1. ACEIs and ARBs can be safely used during pregnancy.
   1. True
   2. **False**
2. Excessive hair growth is a side effect of some antihypertensive medications
   1. **True**
   2. False
3. Some antihypertensives may not show their maximum effect if the patient does not control his salt intake.
   1. **True**
   2. False
4. Once there is a side effect with an antihypertensive medicine, it must be discontinued.
   1. True
   2. **False**
5. Which of the antihypertensive medicines can worsen gout?
   1. Calcium channel blockers (CCBs)
   2. Angiotensin receptor blockers (ARBs)
   3. Beta-blockers
   4. **Thiazide and thiazide-like group**
6. Erectile dysfunction is rare with antihypertensive medicines:
   1. **True**
   2. False
