# Supplementary material for: Implementation of the Extension for Community Healthcare Outcomes Model for Hypertension Education of Frontline Health Care Workers in the Federal Capital Territory, Nigeria: Explanatory Sequential Mixed Methods Evaluation
Source: J Med Internet Res. 2025 Apr 24;27:e66351. doi: 10.2196/66351 (PMC12062761; doi:10.2196/66351)
Supplement: Multimedia Appendix 5 [file jmir_v27i1e66351_app5.pdf]

HTN ECHO Program

Thank you for participating in the HTN ECHO session on:

Date: April 13, 2023Topic: Complications of Antihypertensive Drug TherapyPresenters: Prof. Emmanuel Ejim, Prof. Ifeoma Ulasi & Dr. Abimbola OpadeyiKeeping in mind the information you learned during that session, please respond to the following questions.

Completion Date

____

Please share some information on your role in the healthcare system.

In what country are you located?

- ◯ Nigeria
- ◯ Somewhere else

In what country do you work?

____

Do you work in the Federal Capital Territory?

- ◯ Yes
- ◯ No

In what state do you work?

____

In which council area do you work?

- ◯ Abaji (01)
- ◯ AMAC (02)
- ◯ Bwari (03)
- ◯ Gwagwalada (04)
- ◯ Kuje (05)
- ◯ Kwali (06)

In which Abaji PHC do you work?

- ◯ AGYANA PHC
- ◯ CENTRAL PHC
- ◯ GAWU PHC
- ◯ LOW COST PHC
- ◯ NAHARATI PHC
- ◯ NEW TOWNSHIP CLINIC
- ◯ RIMBA PHC
- ◯ YABA PHC
- ◯ Another PHC
- ◯ Another type of health facility

In which AMAC PHC do you work?	◯ APO PHC<br>◯ DAKWA PHC<br>◯ GBAGARAPE PHC<br>◯ GIDAN-MANGORO CPHC<br>◯ GOSA PHC<br>◯ GWAGWA PHC<br>◯ JIKWOYI PHC<br>◯ KAGINI PHC<br>◯ KARONMAJIGI PHC<br>◯ KARSHI PHC<br>◯ KARU PHC<br>◯ KUCHINGORO PHC<br>◯ LUGBE PHC<br>◯ OROZO PHC<br>◯ PYAKASSA PHC<br>◯ Another PHC<br>◯ Another type of health facility
In which Bwari PHC do you work?	◯ BYAZHIN PHC<br>◯ DEIDEI CPHC<br>◯ KOGO PHC<br>◯ MPAPE PHC<br>◯ SABONGARI PHC<br>◯ SHERE PHC<br>◯ USHAFA PHC<br>◯ PHC KAWU<br>◯ Another PHC<br>◯ Another type of health facility
In which Gwagwalada PHC do you work?	◯ ANAGADA PHC<br>◯ DAGIRI PHC<br>◯ DOBI PHC<br>◯ DUKPA PHC<br>◯ GTC PHC<br>◯ GWAKO PHC<br>◯ OLD KUTUNKU PHC<br>◯ PAIKON PHC<br>◯ RAFIN-ZURFI PHC<br>◯ YIMI PHC<br>◯ ZUBA PHC<br>◯ Another PHC<br>◯ Another type of health facility
In which Kuje PHC do you work?	◯ CHUKUKU PHC<br>◯ GAUBE PHC<br>◯ GUDUNKARYA PHC<br>◯ GWARGWADA PHC<br>◯ KIYI PHC<br>◯ KUJE PHC<br>◯ KUJEKWA PHC<br>◯ PAGI PHC<br>◯ Another PHC<br>◯ Another type of health facility

In which Kwali PHC do you work?	◯ ASHARA PHC<br>◯ DABI BAKO PHC<br>◯ DAFA PHC<br>◯ GOMANI PHC<br>◯ KWALI PHC<br>◯ KWAITA HAUSA PHC<br>◯ KWAITA MODEL PHC<br>◯ PETTI PHC<br>◯ WAKO PHC<br>◯ YANGOJI PHC<br>◯ Another PHC<br>◯ Another type of health facility
Were you able to join the last ECHO session?	◯ Yes, I joined the live Zoom session<br>◯ Yes, I watched the livestream<br>◯ Yes, I watched a recording<br>◯ No
Do you provide patient care?	◯ Yes<br>◯ No

Please respond to the following questions, keeping in mind the information you learned during the last session.

In the last 30-days, did you use any of the information you learned in the last ECHO session to care for patients with high blood pressure?	◯ Yes<br>◯ No
Please describe how you used the information you learned in the last ECHO session	________________________
In the last 30-days, did a patient with high blood pressure ask you any questions about their high blood pressure that you could not answer?	◯ Yes<br>◯ No
Please describe the patients' question(s)	________________________
In the last 30 days, were you ever in a situation where you were unsure of how to tend to a patient with high blood pressure?	◯ Yes<br>◯ No
Please describe the situation	________________________
