# Supplementary material for: Implementation of the Extension for Community Healthcare Outcomes Model for Hypertension Education of Frontline Health Care Workers in the Federal Capital Territory, Nigeria: Explanatory Sequential Mixed Methods Evaluation
Source: J Med Internet Res. 2025 Apr 24;27:e66351. doi: 10.2196/66351 (PMC12062761; doi:10.2196/66351)
Supplement: Multimedia Appendix 7 [file jmir_v27i1e66351_app7.docx]

**Table S2.** Characteristics of frontline healthcare worker focus group discussion participants from 12 Hypertension Treatment in Nigeria Program primary health centers.

| **Characteristic, No. (%)** | **No. Responses** | **All**  **(n=31)** | **FGD1**  **(n=4)** | **FGD2**  **(n=4)** | **FGD3**  **(n=7)** | **FGD4**  **(n=6)** | **FGD5**  **(n=4)** | **FGD6**  **(n=6)** |
| --- | --- | --- | --- | --- | --- | --- | --- | --- |
| Age, median (range) | 30 | 40 (26-57) | 43 (32-47) | 36 (30-40) | 45 (32-50) | 40 (35-46) | 40 (39-42) | 46 (26-57) |
| Female | 30 | 19 (63) | 4 (100) | 2 (50) | 5 (71) | 4 (67) | 1 (33) | 3 (50) |
| Role | 31 |  |  |  |  |  |  |  |
| Community Health Extension Worker |  | 15 (48) | 3 (75) | 2 (50) | 4 (57) | 2 (33) | 2 (50) | 2 (33) |
| Record Officer |  | 2 (6) | 0 (0) | 0 (0) | 0 (0) | 1 (17) | 0 (0) | 1 (17) |
| Facility Manager |  | 1 (3) | 0 (0) | 0 (0) | 0 (0) | 1 (17) | 0 (0) | 0 (0) |
| Laboratory Technician |  | 3 (10) | 0 (0) | 1 (25) | 0 (0) | 0 (0) | 1 (25) | 1 (17) |
| Nurse |  | 7 (23) | 1 (25) | 0 (0) | 3 (43) | 0 (0) | 1 (25) | 2 (33) |
| Pharmacy Focal Person |  | 3 (10) | 0 (0) | 1 (25) | 0 (0) | 2 (33) | 0 (0) | 0 (0) |
| Years of Experience, median (range) | 30 | 15 (3-25) | 16 (5-20) | 9 (5-12) | 15 (7-23) | 15 (7-21) | 15 (15-20) | 13 (3-25) |
| Involved in the Hypertension Treatment in Nigeria Program | 31 | 31 (100) | 4 (100) | 4 (100) | 7 (100) | 6 (100) | 4 (100) | 6 (100) |
| Treats or Manages Hypertension | 31 | 31 (100) | 4 (100) | 4 (100) | 7 (100) | 6 (100) | 4 (100) | 6 (100) |
| Number of Sessions Participated (range) | 30 | 3 (1-7) | 4 (3-6) | 6 (4-7) | 1 (1-3) | 4 (3-6) | 4 (1-4) | 2 (1-7) |
