# Supplementary material for: Implementation of the Extension for Community Healthcare Outcomes Model for Hypertension Education of Frontline Health Care Workers in the Federal Capital Territory, Nigeria: Explanatory Sequential Mixed Methods Evaluation
Source: J Med Internet Res. 2025 Apr 24;27:e66351. doi: 10.2196/66351 (PMC12062761; doi:10.2196/66351)
Supplement: Multimedia Appendix 8 [file jmir_v27i1e66351_app8.docx]

**Table S3.** Reaction surveys from live participants in each hypertension ECHO session.

| **Question, No. (%)^1^** | **Session** | | | | | | | ***P*-Value^2^** |
| --- | --- | --- | --- | --- | --- | --- | --- | --- |
|  | **1**  **(n=49)** | **2**  **(n=39)** | **3**  **(n=37)** | **4**  **(n=31)** | **5**  **(n=43)** | **6**  **(n=42)** | **7**  **(n=30)** |  |
| The training met my expectations | 48 (98) | 39 (100) | 37 (100) | 30 (97) | 43 (100) | 42 (100) | 30 (100) | .52 |
| The training will be useful to me for treating patients with high blood pressure | 49 (100) | 39 (100) | 36 (97) | 31 (100) | 43 (100) | 42 (100) | 30 (100) | .39 |
| Was the length of the session appropriate? |  |  |  |  |  |  |  | .21 |
| Yes, the length was just right | 36 (74) | 28 (72) | 33 (89) | 28 (90) | 34 (79) | 32 (76) | 25 (83) |  |
| No, it should have been shorter | 11 (22) | 7 (18) | 3 (8) | 1 (3) | 9 (21) | 8 (19) | 5 (17) |  |
| No, it should have been longer | 2 (4) | 4 (10) | 1 (3) | 2 (7) | 0 (0) | 2 (5) | 0 (0) |  |
| How helpful was the material presented by the speakers? |  |  |  |  |  |  |  | .039 |
| Very helpful | 45 (92) | 37 (95) | 35 (95) | 22 (71) | 40 (93) | 40 (95) | 28 (93) |  |
| Somewhat helpful | 2 (4) | 2 (5) | 2 (5) | 9 (29) | 3 (7) | 2 (5) | 2 (7) |  |
| Neither helpful nor unhelpful | 1 (2) | 0 (0) | 0 (0) | 0 (0) | 0 (0) | 0 (0) | 0 (0) |  |
| Somewhat unhelpful | 0 (0) | 0 (0) | 0 (0) | 0 (0) | 0 (0) | 0 (0) | 0 (0) |  |
| Unhelpful | 1 (2) | 0 (0) | 0 (0) | 0 (0) | 0 (0) | 0 (0) | 0 (0) |  |
| How helpful was the case presentation? |  |  |  |  |  |  |  | .39 |
| Very helpful | 40 (82) | 36 (92) | 32 (87) | 24 (77) | 40 (93) | 34 (81) | 23 (77) |  |
| Somewhat helpful | 8 (16) | 3 (8) | 5 (14) | 6 (19) | 3 (7) | 8 (19) | 6 (20) |  |
| Neither helpful nor unhelpful | 1 (2) | 0 (0) | 0 (0) | 0 (0) | 0 (0) | 0 (0) | 1 (3) |  |
| Somewhat unhelpful | 0 (0) | 0 (0) | 0 (0) | 0 (0) | 0 (0) | 0 (0) | 0 (0) |  |
| Unhelpful | 0 (0) | 0 (0) | 0 (0) | 1 (3) | 0 (0) | 0 (0) | 0 (0) |  |

^1^Proportions are from among those who responded.

^2^Chi-squared test
