# Supplementary material for: Implementation of the Extension for Community Healthcare Outcomes Model for Hypertension Education of Frontline Health Care Workers in the Federal Capital Territory, Nigeria: Explanatory Sequential Mixed Methods Evaluation
Source: J Med Internet Res. 2025 Apr 24;27:e66351. doi: 10.2196/66351 (PMC12062761; doi:10.2196/66351)
Supplement: Multimedia Appendix 9 [file jmir_v27i1e66351_app9.docx]

**Table S4.** Pre- and posttest knowledge surveys from each hypertension ECHO session among all participants who responded.

| **No. Correct (%)** | **Session 1** | | **Session 2** | | **Session 3** | | **Session 4** | | **Session 5** | | **Session 6** | | **Session 7** | |
| --- | --- | --- | --- | --- | --- | --- | --- | --- | --- | --- | --- | --- | --- | --- |
|  | **Pre**  **N=57** | **Post**  **N=43** | **Pre**  **N=48** | **Post**  **N=43** | **Pre**  **N=40** | **Post**  **N=36** | **Pre**  **N=30** | **Post**  **N=35** | **Pre**  **N=46** | **Post**  **N=55** | **Pre**  **N=50** | **Post**  **N=54** | **Pre**  **N=44** | **Post**  **N=33** |
| Question 1 | 49 (86) | 37 (86) | 38 (79) | 41 (95) | 13 (33) | 22 (61) | 23 (77) | 25 (71) | 46 (100) | 54 (98) | 42 (84) | 47 (87) | 31 (70) | 28 (85) |
| Question 2 | 37 (65) | 34 (79) | 35 (73) | 32 (74) | 36 (90) | 32 (89) | 8 (27) | 13 (37) | 40 (87) | 47 (85) | 30 (60) | 42 (78) | 33 (75) | 29 (88) |
| Question 3 | 47 (82) | 42 (98) | 26 (54) | 14 (33) | 29 (73) | 30 (83) | 17 (57) | 21 (60) | 29 (63) | 45 (82) | 14 (28) | 25 (46) | 27 (61) | 28 (85) |
| Question 4 | 27 (47) | 25 (58) | 23 (48) | 30 (70) | 28 (70) | 22 (61) | 9 (30) | 11 (31) | 24 (52) | 28 (51) | 35 (70) | 43 (80) | 18 (41) | 11 (33) |
| Question 5 | 51 (89) | 41 (95) | 41 (85) | 39 (91) | 21 (53) | 22 (61) | 4 (13) | 12 (34) | 32 (70) | 46 (84) | 35 (70) | 41 (76) | 38 (86) | 27 (82) |
| Question 6 | 20 (35) | 18 (42) | 7 (15) | 3 (7) | 25 (63) | 31 (86) | 15 (50) | 18 (51) | 18 (39) | 31 (56) | 38 (76) | 45 (83) | 22 (50) | 15 (45) |
| Question 7 | 51 (89) | 40 (93) | 22 (46) | 27 (63) | 18 (45) | 21 (58) | 15 (50) | 23 (66) | 36 (78) | 42 (76) | 13 (26) | 14 (26) | 18 (41) | 14 (42) |
| Question 8 | 29 (51) | 31 (71) | 26 (54) | 28 (65) | 22 (55) | 28 (78) | 2 (7) | 8 (23) | 22 (48) | 32 (58) | 29 (58) | 34 (63) | 17 (39) | 7 (21) |
| Question 9 |  |  |  |  | 26 (65) | 25 (69) |  |  |  |  |  |  |  |  |
| Question 10 |  |  |  |  | 31 (78) | 29 (81) |  |  |  |  |  |  |  |  |
| Average Score (%) | 5.5 (68.2) | 6.2 (77.9) | 4.5  (56.8) | 5.0  (62.2) | 6.2  (62.3) | 7.3  (72.8) | 3.1  (38.8) | 3.7  (46.8) | 5.4  (67.1) | 5.9  (73.9) | 4.7  (59.0) | 5.4  (67.4) | 4.6  (58.0) | 4.8  (60.2) |
