# Supplementary material for: Implementation of the Extension for Community Healthcare Outcomes Model for Hypertension Education of Frontline Health Care Workers in the Federal Capital Territory, Nigeria: Explanatory Sequential Mixed Methods Evaluation
Source: J Med Internet Res. 2025 Apr 24;27:e66351. doi: 10.2196/66351 (PMC12062761; doi:10.2196/66351)
Supplement: Multimedia Appendix 10 [file jmir_v27i1e66351_app10.docx]

**Table S5.** Pre- and posttest knowledge surveys from each hypertension ECHO session among participants who responded to both the pre- and postquizzes.

| No. Correct (%) | **Session 1** | | **Session 2** | | **Session 3** | | **Session 4** | | **Session 5** | | **Session 6** | | **Session 7** | | |
| --- | --- | --- | --- | --- | --- | --- | --- | --- | --- | --- | --- | --- | --- | --- | --- |
|  | **Pre**  **N=29** | **Post**  **N=29** | **Pre**  **N=22** | **Post**  **N=22** | **Pre**  **N=15** | **Post**  **N=15** | **Pre**  **N=16** | **Post**  **N=16** | **Pre**  **N=28** | **Post**  **N=28** | **Pre**  **N=32** | **Post**  **N=32** | | **Pre**  **N=23** | **Post**  **N=23** |
| Question 1 | 26 (90) | 25 (86) | 15 (68) | 21 (95) | 2 (13) | 10 (67) | 12 (75) | 11 (69) | 28 (100) | 28 (100) | 26 (81) | 28 (88) | | 18 (78) | 18 (78) |
| Question 2 | 18 (62) | 24 (83) | 15 (68) | 16 (73) | 13 (87) | 13 (87) | 5 (31) | 8 (50) | 24 (86) | 24 (86) | 23 (72) | 25 (78) | | 16 (70) | 20 (87) |
| Question 3 | 25 (86) | 28 (97) | 14 (64) | 9 (41) | 12 (80) | 11 (73) | 8 (50) | 12 (75) | 17 (61) | 22 (79) | 10 (31) | 14 (44) | | 16 (70) | 20 (87) |
| Question 4 | 13 (45) | 16 (55) | 7 (32) | 18 (82) | 12 (80) | 11 (73) | 7 (44) | 3 (19) | 13 (46) | 15 (54) | 20 (63) | 27 (84) | | 10 (43) | 6 (26) |
| Question 5 | 27 (93) | 27 (93) | 19 (86) | 19 (86) | 7 (47) | 10 (67) | 1 (6) | 9 (56) | 18 (64) | 24 (86) | 22 (69) | 27 (84) | | 20 (87) | 19 (83) |
| Question 6 | 10 (34) | 13 (45) | 2 (9) | 1 (5) | 9 (60) | 11 (73) | 10 (63) | 8 (50) | 14 (50) | 13 (46) | 24 (75) | 27 (84) | | 11 (48) | 9 (39) |
| Question 7 | 27 (93) | 29 (100) | 13 (59) | 15 (68) | 5 (33) | 9 (60) | 7 (44) | 10 (63) | 22 (79) | 24 (86) | 11 (34) | 12 (38) | | 9 (39) | 12 (52) |
| Question 8 | 12 (41) | 22 (76) | 12 (55) | 14 (64) | 9 (60) | 12 (80) | 1 (6) | 4 (25) | 13 (46) | 17 (61) | 19 (59) | 22 (69) | | 10 (43) | 4 (17) |
| Question 9 |  |  |  |  | 10 (67) | 11 (73) |  |  |  |  |  |  | |  |  |
| Question 10 |  |  |  |  | 11 (73) | 12 (80) |  |  |  |  |  |  | |  |  |
| Average Score (%) | 5.4 (68.1) | 6.3  (79.3) | 4.4  (55.1) | 5.1  (64.2) | 6.0  (60.0) | 7.3  (73.3) | 3.2  (39.8) | 4.1  (50.8) | 5.3  (66.5) | 6.0  (74.6) | 4.8  (60.5) | 5.7  (71.1) | | 4.8  (59.8) | 4.7  (58.7) |
| P-Value^*^ | <0.001 | | 0.004 | | 0.013 | | 0.042 | | 0.06 | | 0.008 | | | 0.64 | |

*P-value is from either Wilcoxon signed-rank test or paired t-test (session 6 only) based on parametricity of the data.
