# Supplementary material for: Implementation of the Extension for Community Healthcare Outcomes Model for Hypertension Education of Frontline Health Care Workers in the Federal Capital Territory, Nigeria: Explanatory Sequential Mixed Methods Evaluation
Source: J Med Internet Res. 2025 Apr 24;27:e66351. doi: 10.2196/66351 (PMC12062761; doi:10.2196/66351)
Supplement: Multimedia Appendix 11 [file jmir_v27i1e66351_app11.docx]

**Table S6.** Participant feedback on use of information following each ECHO session among healthcare workers at 12 Hypertension Treatment in Nigeria Program primary health centres.

| **Characteristic, No. (%)^1^** | **Session** | | | | | | | ***P*-value^2^** |
| --- | --- | --- | --- | --- | --- | --- | --- | --- |
|  | **1**  **(n=49)** | **2**  **(n=30)** | **3**  **(n=19)** | **4**  **(n=18)** | **5**  **(n=9)** | **6**  **(n=13)** | **7**  **(n=8)** |  |
| Days elapsed since ECHO session, median (range) | 26 (22-31) | 29 (28-38) | 21 (20-27) | 91 (89-96) | 25 (22-27) | 51 (20-55) | 60 (34-78) | <.0001 |
| Area Council |  |  |  |  |  |  |  | <.0001 |
| Abaji | 4 (8) | 3 (10) | 3 (16) | 1 (6) | 3 (33) | 2 (15) | 1 (13) |  |
| AMAC | 5 (10) | 3 (10) | 9 (47) | 0 (0) | 0 (0) | 1 (8) | 0 (0) |  |
| Bwari | 14 (29) | 4 (14) | 1 (5) | 1 (6) | 0 (0) | 2 (15) | 0 (0) |  |
| Gwagwalada | 9 (19) | 2 (7) | 4 (21) | 4 (24) | 2 (22) | 1 (8) | 2 (25) |  |
| Kuje | 14 (29) | 11 (38) | 1 (5) | 2 (12) | 1 (11) | 1 (8) | 1 (13) |  |
| Kwali | 2 (4) | 6 (21) | 1 (5) | 9 (53) | 3 (33) | 6 (46) | 4 (50) |  |
| Able to join the ECHO Session |  |  |  |  |  |  |  | .049 |
| Yes, Live Zoom | 28 (57) | 9 (30) | 15 (79) | 9 (50) | 7 (78) | 5 (38) | 5 (63) |  |
| Yes, Livestream | 1 (2) | 2 (7) | 0 (0) | 0 (0) | 0 (0) | 0 (0) | 1 (13) |  |
| Yes, a recording | 4 (8) | 2 (7) | 2 (11) | 0 (0) | 0 (0) | 3 (23) | 0 (0) |  |
| No | 16 (33) | 17 (57) | 2 (11) | 9 (50 | 2 (22) | 5 (38) | 2 (25) |  |
| Provide Patient Care | 47 (98) | 29 (97) | 13 (100) | 18 (100) | 9 (100) | 13 (100) | 8 (100) | .92 |
| **Individuals Who Provide Patient Care and Participated in the Last ECHO Session, N** | 31 | 12 | 17 | 9 | 7 | 8 | 6 |  |
| In the past 30-days |  |  |  |  |  |  |  |  |
| Used information from the last ECHO session | 30 (97) | 9 (75) | 15 (88) | 7 (78) | 5 (71) | 7 (88) | 6 (100) | .27 |
| Had a patient question about hypertension that they could not answer | 5 (17) | 2 (17) | 3 (18) | 1 (11) | 2 (29) | 1 (13) | 0 (0) | .89 |
| Were in a situation where they were unsure how to care for a hypertensive patient | 4 (13) | 1 (8) | 0 (0) | 1 (11) | 2 (29) | 1 (13) | 0 (0) | .47 |

^1^Proportions are from among those who responded.

^2^Kruskal-Wallis or Chi-squared test
